# B Cell Subsets in Colombian Adults with Predominantly Antibody Deficiencies, Bronchiectasis or Recurrent Pneumonia

**DOI:** 10.3390/arm90040035

**Published:** 2022-07-27

**Authors:** Sebastian Giraldo-Ocampo, Anilza Bonelo, Andres F. Zea-Vera

**Affiliations:** 1 VIREM Research Group, Departamento de Microbiología, Universidad del Valle, Cl. 4b #36b-37, Cali 760032, Colombia; sebastian.giraldo.ocampo@correounivalle.edu.co (S.G.-O.); anilza.bonelo@correounivalle.edu.co (A.B.); 2 Hospital Universitario del Valle, Cl. 5 #36-08, Cali 760032, Colombia

**Keywords:** B cell subsets, flow cytometry, predominantly antibody deficiencies, bronchiectasis, recurrent pneumonia, inborn errors of immunity, primary immunodeficiencies, PIDD, adults, Colombia

## Abstract

**Highlights:**

- Flow cytometry evaluation of B cells subsets in peripheral blood successfully recognizes subjects predominantly with antibodies deficiencies.
- Memory B cell populations (especially switched memory B cells) are decreased in subjects predominantly with antibodies deficiencies.
- CD8+ T cells and NK cells are affected in subjects with common variable immunodeficiency.
- Patients with non-cystic fibrosis bronchiectasis or recurrent pneumonia exhibit a reduction in naive B cells.

**Abstract:**

Aim: To evaluate and describe lymphocyte populations’ and B cell subsets’ frequencies in patients presenting with Predominantly antibody deficiencies (PAD) and diagnosed with bronchiectasis or recurrent pneumonia seen in Cali (Colombian Southwest region). Materials and Methods: 16 subjects with PAD, 20 subjects with pulmonary complications (bronchiectasis or recurrent pneumonia) and 20 healthy donors (HD). Controls and probands between 14 and 64 years old, regardless of gender were included. Lymphocyte populations (T, B and NK cells) and B cell subsets were evaluated in peripheral blood mononuclear cells using flow cytometry, T/B/NK reagent and the pre-germinal center antibody panel proposed by the EUROflow consortium were used. EUROclass and the classification proposed by Driessen et al. were implemented. Results: CVID patients exhibited increase absolute numbers of CD8+ T cells and reduce NK cells as compare with HD, other PAD cases or pulmonary complications. PAD B cell subsets were disturbed when compared to the age range-matched healthy donors. Among B cell subsets, the memory B cell compartment was the most affected, especially switched memory B cells. Four participants were classified as B- and two CVID as smB-Trnorm and smB-21low groups according to EUROclass classification. The most frequent patterns proposed by Driessen et al. were B cell production and germinal center defect. Conclusions: B cell subsets, especially memory B cells, are disturbed in PAD patients from Southwestern Colombia. To the best of our knowledge this is the most comprehensive study of B cell subsets in Colombian adults.

## 1. Introduction

Predominantly antibody deficiencies (PAD) are the most prevalent group of the ten categories of inborn errors of immunity currently recognized by the International Union of Immunological Societies, comprising about half of these disorders [1,2]. Among PAD, common variable immunodeficiency (CVID), selective IgA deficiency (SIgAD) and agammaglobulinemia are the most representative disorders, although other disorders are recognized as PAD, such as selective IgM deficiency (SIgMD) and specific antibody deficiency [3,4]. Frequently, patients with PAD, especially with CVID, have an unusual susceptibility to sinopulmonary infections, commonly by encapsulated bacteria, leading to recurrent pneumonia [5]. The recurrent infections may cause different pulmonary complications, non-cystic fibrosis bronchiectasis (NCFB) being the most prevalent in PAD, with a mean of 34% in CVID patients [6]. In turn, the vicious cycle of inflammation and infection of bronchiectasis could contribute to the disturbance of the humoral immune response and therefore to infection susceptibility [7].

The diagnosis criteria for PAD disorders are well established by the European Society for Immunodeficiencies (ESID) and are based on serum immunoglobulin levels, responses against vaccine antigens and no profound alterations in the T cell compartment along with increased susceptibility to infections, autoimmunity, lymphoproliferation, granulomas and/or history of affected family members [8]. Due to alterations in the humoral adaptative immune response in PAD patients, the evaluation of B cell subsets could provide relevant information for the characterization of each patient and some clues about the physio-pathological mechanism of the disease [9]. Accordingly, three classification systems have been proposed, based on B cell subsets, that allocate CVID patients in homogeneous groups: Freiburg, Paris and Euroclass [10,11,12]. The group in which a patient is allocated can be associated with certain complications such as autoimmunity, lymphoproliferation or splenomegaly. Among the three classification systems, Euroclass is the one with more B cell subsets and groups incorporated and allows discrimination of CVID patients with non-infectious complications similar to Freiburg and Paris [12]. A fourth classification system was proposed by Driessen et al. [13], in which each patient is allocated in one of five B cell patterns each reflecting a different pathophysiology of the disease.

Currently, published data about immunological assessment in subjects diagnosed with PAD are limited in Colombia. The few studies available were published by the primary immunodeficiency group from Universidad de Antioquia [14]. For this reason, the aim of this study was to evaluate and describe the lymphocyte populations and B cell subsets frequency in patients seen in Cali, who came from different locations from the Colombian Southwest region.

### Clinical Rationale for the Study

B cell subset evaluation is critical for the characterization and classification of patients with predominantly antibody deficiencies. However, so far, no information is available about the frequencies or distribution of these subsets in patients from Cali, Colombia. in this regard, this is the first study showing B cell subsets in subjects with this type of inborn error of immunity in the city.

## 2. Methods

### 2.1. Study Subjects

This is an observational, transversal, analytical and non-interventional study that enrolled patients with predominantly antibody deficiencies, bronchiectasis or recurrent pneumonia.

All cases were referred to the Clinical Immunology Clinic at Universidad del Valle (Cali, Colombia) by pulmonologists, internists or allergists attending to patients from Southwest Colombia (Valle del Cauca, Cauca and Nariño departments) in different medical centers in Cali from 2 January 2019 to 31 March 2020.

Patients enrolled fulfilling the inclusion criteria were: aged ≥ 14 and <65 years, regardless of gender, with bronchiectasis on chest computed tomography (CT) and the clinical syndrome of bronchiectasis (cough, sputum production or recurrent respiratory infections) or with recurrent pneumonia (at least two pneumonias in one year or more than three pneumonias throughout life, with radiographic resolution between episodes). Exclusion criteria included inability to give informed consent, bronchiectasis due to cystic fibrosis, acquired immune defects or secondary immunodeficiencies (chronic myeloid leukemia, multiple myeloma, human immunodeficiency virus (HIV) infection, immunosuppression secondary to drugs). Inborn errors of immunity were defined according to the ESID classification and diagnostic criteria. Peripheral, anticoagulated blood samples with EDTA and clinical data were collected and stored according to the institutional review board protocols.

The inborn error of immunity diagnosis was corroborated by the physician from the study (clinical immunologist). In case of discrepancy, a third physician was contacted. PAD were defined according to the ESID criteria [8], bronchiectasis according to the British Thoracic Society guideline for non-CF bronchiectasis [15] and recurrent pneumonia as two episodes of pneumonia in one year or three in the lifetime with radiography resolution between episodes [16].

The study was approved by the institutional committee of human ethical review of the Universidad del Valle, ID: 010-019R and by the ethics committee of Hospital Universitario del Valle “Evaristo Garcia”, ID: 050-2019. Informed consent was obtained from all patients and healthy donors. This study was performed in line with the principles of the Declaration of Helsinki.

### 2.2. Isolation of Peripheral Blood Mononuclear Cells (PBMC) and Leucocytes Evaluation

PBMCs were isolated by density gradient centrifugation using Ficoll-Paque (Sigma-Aldrich, St. Louis, MO, USA). Briefly, total blood was diluted in 10 mL of RPMI 1640 and centrifuged at 1200 RPM with 3 mL of Ficoll-Paque. The upper layer containing the plasma was removed and the PMBC fraction was transferred into a new 15 mL tube and washed twice with 10 mL RPMI 1640. Cell viability and counting were performed using trypan blue staining. PBMC were cryopreserved in liquid nitrogen in freezing medium (40% fetal bovine serum (FBS)/10% DMSO) until the analysis. A total of 500 uL of blood was used for the evaluation of white blood cells by hemogram analysis.

PBMC were thawed in a water bath at 37 °C and then FBS was added drop by drop; then the cells were transferred to a sterile 15 mL Falcon tube, washed twice with 10 mL of RPMI and 10% FBS, and centrifuged at 1200 RPMI for 10 min. Finally, the cellular pellet was reconstituted with 2 mL of FACS Buffer (PBS 1X, 10 mM EDTA and 5% FBS). Cell viability and count were performed as described above. Only samples with a viability index greater than 80% were evaluated.

### 2.3. Flow Cytometric Analysis

The evaluation of lymphocytes populations was performed using 10 µL of the 6-color TBNK reactive from Becton Dickinson (BD) in a final volume of 60 µL. A total of 2.5 × 10^5^ cells were stained and incubated for 15 min at room temperature and then all of the events were acquired in the cytometer. The identification of the populations was as follow: CD4^+^ T cells (CD45^+^CD3^+^CD4^+^), CD8^+^ T cells (CD45^+^CD3^+^CD8^+^), double-negative T cells (CD45^+^CD3^+^CD4^−^CD8^−^), double-positive T cells (CD45^+^CD3^+^CD4^+^CD8^+^), B cells (CD45^+^CD3^−^CD19^+^) and NK cells (CD45^+^CD3^−^(CD16-CD56)^+^).

Immunophenotyping of B cell subsets was performed in PBMCs using the pre-germinal center (pre-GC) antibody panel proposed by the EuroFlow Consortium [17]: CD19- PECy7 (clone J3-119) from Beckman Coulter; CD5-PE (clone UCHT-2), SmIgD-PerCPCy5.5 (clone IA6-2) and SmIgM-BV510 (clone MHM-88) from Biolegend; CD21-APC (clone B-ly4), CD27-BV421 (clone M-T271) and CD38-HB7 from BD Biosciences. The antibody volumes were 2.5, 2.5, 0.75, 0.65, 5, 0.5 and 2.5 µL, respectively, in a final volume of 100 µL. The cells were incubated for 30 min at room temperature and then the antibodies in the supernatant were washed with FACS buffer. A minimum of 7.5 × 10^5^ events/leukocytes were acquired per tube. CompBeads Set Anti-Mouse Ig, κ (BD bioscience) was used for fluorescence compensation.

**Figure 1:**
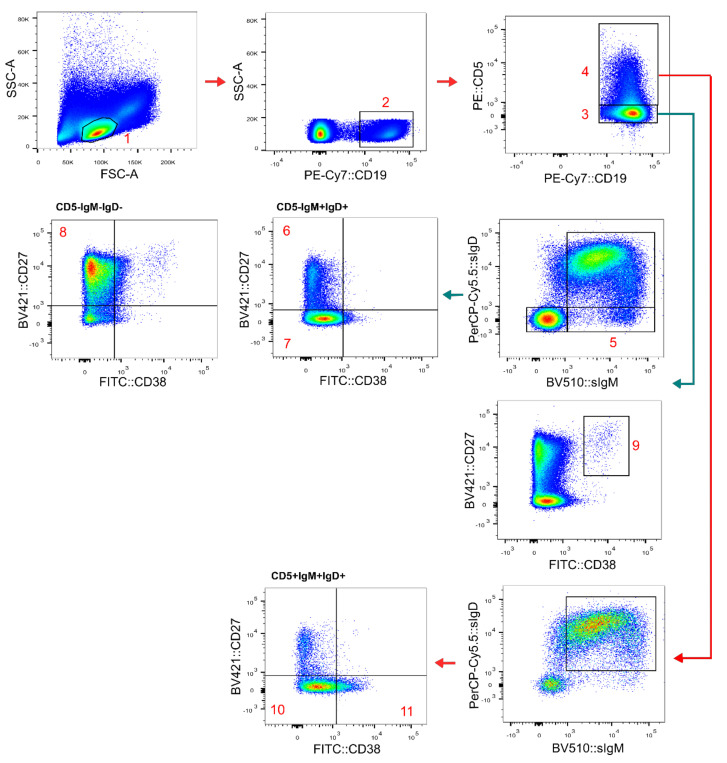
Gating strategy for the main B cell subsets identification. Doublets were excluded (not shown). 1. Lymphocytes; 2. B cells; 3. CD5- B cells; 4. CD5+ B cells; 5. IgM-only memory cells (positive for CD27, not shown); 6. unswitched memory B cells; 7. naive CD5- B cells; 8. switched memory B cells; 9. plasmablasts; 10. naive CD5+ B cells; 11. transitional B cells.

Based on the staining pattern of the above mentioned antibodies and the side (SSC) and forward scatter (FSC) parameters, B cells (CD19^+^ SSC^low^ FSC^low^ lymphocytes) were classified into immature/transitional B lymphocytes (CD5^+^CD27^−^CD38^++^smIgM^++^smIgD^+^); naïve CD5^+^ (CD5^+^CD27^−^CD38^+d^smIgM^+^smIgD^++^) and CD5^−^ (CD5^−^CD27^−^CD38^+d^smIgM^+^ smIgD^++^) B lymphocytes; CD21low B cells (IgM^+^CD21^−^CD38^−^); switched memory B cells (CD5^−^CD27^+/−^CD38^−^smIgM^−^smIgD^−^); and unswitched memory B cells (CD5^−^CD27^+^CD38^−^smIgM^+^ smIgD^++^), plasmablasts (CD5^−^CD27^++^CD38^+++^) and IgM-only memory cells (CD5^−^IgM^+^IgD-CD27^+^) (Figure 1). B cell subsets were also identified using the IgD vs. CD27 approach for the classification of patients according to EUROclass [12].

A negative control (unstained cells) was included for each sample. All assays were performed in a FACSCanto™ II cytometer using FACSDiva (BD) software. Data analysis was performed with Flowjo vX.0. and absolute frequencies were calculated using the dual-platform approach. A decrease or increase in the B cell subsets were respectively defined as a value below the minimum value divide by 1.5 or above the maximum value multiplied by 1.5 of the corresponding age ranges: 14–29, 30–39, 40–50 and more than 50 years old. This approach was used due to the non-normality of the data and to detect an important quantitative alteration in the B cell subset evaluated.

PAD participants were classified according to EUROclass [12] and Driessen et al. [13]. The former defines the following groups: B-, SmB- and SmB+, smB+21norm, smB+21lo, smB-Trnorm, smB-Trhi, smB-21lo and smB-21norm after evaluation of B cells, switched memory B cells, transitional B cells and CD21low B cells. Driessen et al. defined five patterns: 1. B cell production and germinal center defect; 2. early peripheral B cell maturation or survival defect; 3. B cell activation and proliferation defect; 4. germinal center defect; 5. post-germinal center defect.

### 2.4. Statistical Analysis

Statistical analysis was performed with the R programing language in RStudio software version 4.1.0. The median and interquartile range of variables are shown. Mann–Whitney and Kruskal–Wallis tests were used to compare groups with continuous outcomes. Fisher’s exact test was used for categorical variables. Two-sided *p* < 0.05 was used to define statistical significance.

## 3. Results

### 3.1. Subjects Characteristics

We enrolled 56 participants: 16 patients with predominantly antibody deficiencies (PAD) and NCFB or recurrent pneumonia, 20 patients with NCFB or recurrent pneumonia without humoral deficiencies (pulmonary complications (PC) group) and 20 gender and age-matched healthy controls (HD group). The PAD group included nine CVID patients, three with SIgAD, one with both SIgAD and IgG subclass deficiency (IgG2 and IgG4), two with SIgMD and one with unclassified antibody deficiency. The pulmonary complications (PC) group had six subjects with bronchiectasis, seven with recurrent pneumonia and seven with both conditions. However, one patient with recurrent pneumonia was excluded from the analysis due to a myeloma diagnosis after the inclusion in the study.

The median ages for the PAD, pulmonary complications and healthy donors’ groups were 35 (IQR: 24–52), 39 (IQR: 29–54) and 36 (IQR: 28–42) years old, respectively, with no difference between groups (*p*-value = 0.99). The proportions of males and females were similar between groups (*p*-value = 0.8) and predominantly of mestizo ethnicity. The sociodemographic information, as well as data about exposure to biomass, wood smoke and tobacco, is depicted in Table 1. The median delay in the PAD diagnosis was 11 (IQR: 6–16) years. The etiology of the bronchiectasis and/or recurrent pneumonia of the participants within the pulmonary complications group were predominantly post-infectious (55%). Other etiologies included asthma, primary ciliary dyskinesia, gastroesophageal reflux, idiopathic and one case with autoimmunity.

For analysis purposes, immunodeficient patients were divided into two groups: CVID and other PAD. The median ages of symptoms’ onset for CVID and other PAD were 19 (IQR: 9–27) and 33 (IQR: 10–38) years old, respectively, with no statistical differences (*p* = 0.8). However, differences were found in diagnosis delay between these groups (*p* = 0.04), with a median of delay, in years, of 6 (IQR: 6–11) in CVID vs. 17 (IQR: 14–28) in other PAD.

Out of the sixteen patients with PAD, nine (56%) had bronchiectasis and recurrent pneumonia (six CVID, two SIgAD and one SIgMD), six (38%) had only recurrent pneumonia (three CVID, one with a non-specified reduction in IgG, one SIgAD and one SIgMD) and one participant, with SIgAD, was diagnosed only with bronchiectasis. However, no differences were detected in the distribution of these lung pathologies between CIVD and other PADs (*p* = 0.4).

### 3.2. Peripheral Blood Lymphocyte Populations

**Figure 2:**
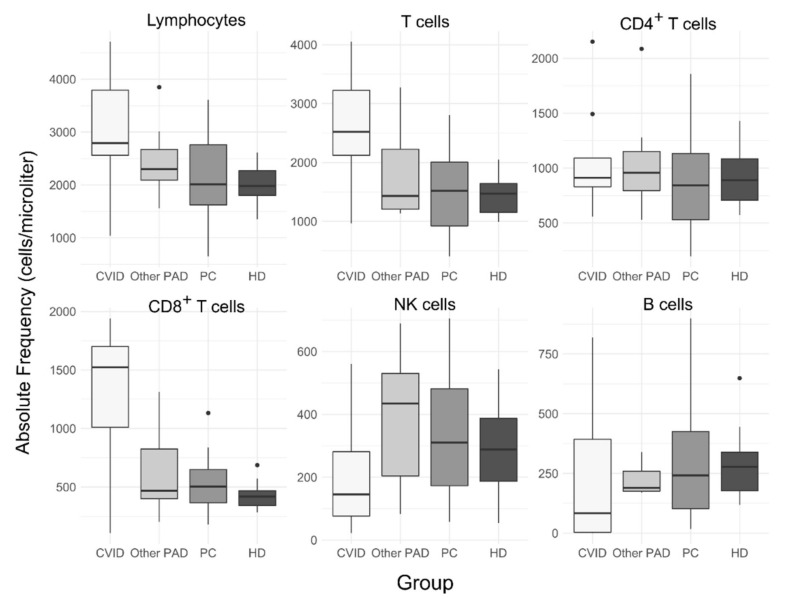
Absolute frequency of peripheral blood lymphocyte populations. Absolute frequency is expressed as cells per microliter of blood. Other PAD: patients with SIgAD, SIgMD or unclassified antibody deficiency; PC: patients with bronchiectasis and/or recurrent pneumonia; HD: healthy donors.

The medians of lymphocytes’ absolute counts were higher in CVID patients compared to the PC, HD and other PAD groups, with 2795 cells/µL, but with no statistical significance between groups (*p* = 0.05) (Figure 2). CVID participants had higher absolute counts of T cells and CD8+ T cells compared to healthy donors and pulmonary complications groups (*p* < 0.05) as well as a lower relative frequency of NK cells compared to these groups, with 5% (IQR: 3–7%) vs. 14% (IQR: 10–17%) and 16% (IQR: 9–20%), respectively, of the total lymphocytes’ frequency. Furthermore, CVID patients had a higher median of T cells relative frequency with 90% (86–93%) compared to the HD, PC and other PAD groups (*p* < 0.05), a lower median of double-negative T cells compared to the PC group and a lower median of the CD4:CD8 ratio compared to healthy donors (*p* < 0.05).

### 3.3. B Cell Subsets

**Figure 3:**
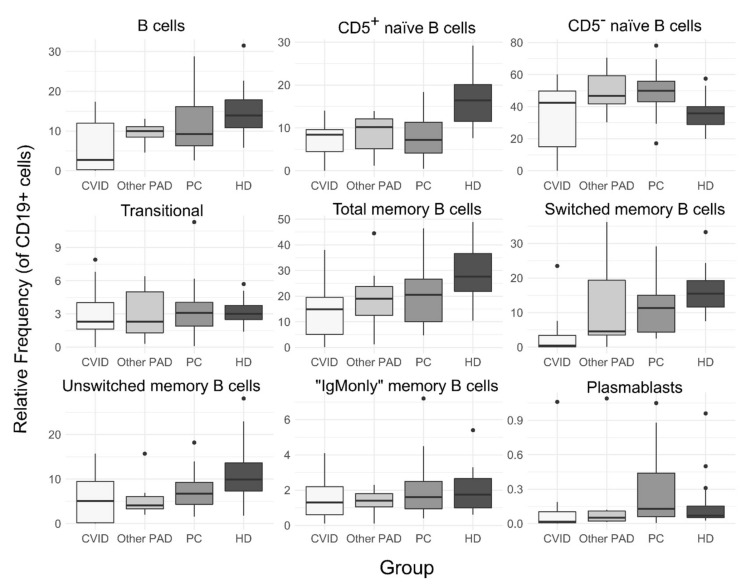
Frequency of B cell subsets as a percentage of B cells (CD19+). Other PAD: patients with SIgAD, SIgMD or unclassified antibody deficiency; PC: patients with bronchiectasis and/or recurrent pneumonia; HD: healthy donors.

There were no differences in B cells’ relative frequency or absolute counts between the four groups (CVID, other PAD, PC and HD); however, CVID participants tended to have a lower median of B cell frequency and absolute counts compared to the other groups (Figure 3), with four out of nine CVID patients with less than one percent of B cells. The relative frequency of memory B cells (CD19+CD27+) was similar between the four groups (*p* > 0.05); however, absolute counts were reduced in CVID patients compared to healthy donors, with 15 (IQR: 1–36) vs. 65 (53–82) cells/µL, respectively. Within this compartment, only switched memory B cells (CD27+smIgM-smIgD-) showed significant variations between groups; the relative frequency of this subset was reduced in CVID patients compared to the pulmonary complications and healthy donor groups (*p* > 0.05) but not to the other PAD group (Figure 2). Similar to the above, the absolute counts of switched memory B cells showed a reduction in CVID patients compared to the PC and HD groups but also between the HD and PC groups; the absolute medians were 1.7 (0.07–4.5), 17 (7–37) and 43 (31–46) cells/µL for the CVID, PC and HD groups, respectively. There were no differences between unswitched and “IgMonly” memory B cells between groups. Similar to the finding of switched memory B cells, the absolute counts of plasmablasts were lower in CVID subjects compared to the PC and HD groups (*p* < 0.05).

Other B cell subsets did not show any differences in relative nor absolute frequency between CVID or other PAD participants and the HD or PC groups. However, the median of CD5^+^ naïve B cells’ relative frequency was higher in healthy donors compared to all other groups (*p* < 0.05) and the median of the relative of CD5^−^ naïve B cells was lower in HD compared to the PC group (*p* < 0.05) (Figure 2).

### 3.4. Alteration of B Cell Subsets Frequencies and Classification of PAD Participants

As mentioned above, four CVID participants had less than 1% B cells, and therefore most of their absolute counts of B cell subsets were reduced when compared to the age range-matched healthy donors. Regarding the other five CVID subjects, three had a reduction in total and switched memory B cells, one unswitched memory B cells and one had normal levels of these B cell subsets (Table 2). Three out of these five CVID patients also had an increase in CD21low B cells’ absolute frequency and only one had a reduction in the plasmablasts absolute count. Out of the four patients with SIgAD, two had a reduction in switched and unswitched memory B cells, one had a reduction only in switched memory and the other one had an increase in unswitched memory B cells. The two participants with SIgMD had increased relative and/or absolute frequencies of total memory B cells, switched and unswitched memory B cells. Only one PAD participant had an alteration in naïve CD5- B cells and three (one SIgAD and the two with SIgMD) had a reduction in CD5+ naïve B cells. The four CVID participants with less than 1% B cells were classified into the B- EUROclass group and three of them in pattern 1 (B cell production and germinal center defect) of Driessen et al.’s classification. Three CVID participants were classified as smB+21norm and two in the smB- group. Three patients were classified separately as pattern 3 (B cell activation and proliferation defect), pattern 4 (Germinal center defect) and pattern 5 (Post germinal center defect) (Table 2).

## 4. Discussion

Predominantly antibody deficiencies are one of the most complex disorders among inborn errors of immunity, with common variable immunodeficiency (CVID), a highly heterogeneous disorder with largely unknown etiology, being the most complex PAD disorder. This study evaluated the peripheral blood lymphocytes’ population and B cell subsets, which have proven to be useful in the classification of PAD patients [12] and as a first approximation to the probable physiopathology mechanism of these disorders [9,13].

Experimental data have revealed disturbance in the T cells’ compartment in CVID patients; however, data are still limited. Reduction in CD4+ T cells and expansion of CD8+ T cells with a senescence phenotype have been found that alter the CD4:CD8 ratio but without the common presentation of T cell alterations such as viral and fungal opportunistic infections [18,19]. A similar finding was seen in the CVID participants from this study, which showed alterations in the T cell compartment; however, CD4+ T cells’ absolute counts were normal but CD8+ T cells were significantly increased, disturbing the CD4:CD8 ratio when compared to healthy donors, and there was a reduction in double-negative T cells and NK cells’ relative frequencies. Conversely, other PAD group (SIgAD, SIgMD and unclassified antibody deficiency) did not show any differences compared to the PC or HD groups, implying that affected cell populations in non-CVID PAD could be restricted to B cell subsets in the subjects of this study.

B cells are classified into different subsets according to their stage of maturation, their role in immune response and their associated and specific markers, such as CD27 (memory marker), superficial membrane IgM and IgD, among others. Transitional B cells are immature B cells (CD5^+^) that recently emerged from the bone marrow and need to mature in the spleen in order to become a marginal-zone or follicular naïve B cell. In this process, CD5^+^ naïve B cells, also known as pre-naïve B cells due to their phenotype that is similar to transitional and CD5^−^ naïve B cells, may emerge. These pre-naïve B cells have been shown to be increased in autoimmune diseases such as systemic lupus erythematosus [20]. Mature, antigen-inexperienced CD5- naïve B cells are the subset able to respond to antigen stimulation and to differentiate into short- and long-lived plasma cells and memory B cells, which ultimately are the cells that produce antibodies and respond rapidly by differentiating into plasma cells during a re-exposure to the antigen, respectively. Within the memory compartment, switched memory B cells differentiate into IgG, IgA or IgE-producing plasma cells; unswitched or marginal-zone like B cells differentiate into IgM-producing plasma cells and are important for anti-polysaccharide responses, and “IgMonly” memory B cells have been found to increase in some disorders such as hyper-IgM syndrome [21]. Finally, other subsets include plasmablasts, which are cells that are able to produce antibodies, migrate and proliferate but ultimately differentiate into plasma cells and CD21low B cells, a subpopulation with an activated phenotype in autoimmune diseases such as systemic lupus erythematosus or autoreactive cells but with anergic phenotype in rheumatoid arthritis and CVID patients [21,22].

Alteration in B cell subsets has been widely recognized to be present in PAD patients, although CVID patients are the more widely studied [9,14,23,24]. We found that the most frequently altered B cell subsets in PAD participants were the B memory compartment, especially switched memory B cells, and plasmablasts, even in non-CVID PAD patients, when compared to HD group (Figure 3) indicating that these subsets are the most frequently altered, as previously reported in Colombian CVID patients [14]; however, no participant showed alterations neither in the frequency of Naïve CD5^+^ that had been associated with autoimmunity [20] nor the naïve CD5^−^ B cells, as reported in a high percentage of the Colombian CVID patients mentioned in the study above, and 4/9 patients showed less than 1% B cells, suggesting different alterations in B cell subsets between these two geographical areas of Colombia, although this comparison has to be taken with caution as different markers and gating strategies were used for the identification of B cell subsets. Moreover, alterations were found in other subsets such as transitional and unswitched memory B cells, especially when compared to the age-associated distribution of normal B cells (Table 2), highlighting the importance of the comparison to healthy donors with a similar age to the patients. Furthermore, one CVD patient had no reduction in switched or unswitched memory B cells, which implies a different alteration pattern.

Accordingly, the EUROclass classification of this patient did not classify them in a group associated with non-infectious complications, and the classification of Driessen et al. [13] classified them in the pattern of post-germinal center defect, although most of the CVID participants were classified as pattern 1 (B cell production and germinal center defect) and pattern 3 (B cell activation and proliferation defect). SIgAD participants showed similar alterations to CVID individuals, implying a possible similar pathological mechanism, and two out of the four participants had B cell activation and proliferation defects. On the other hand, SIgMD showed increased numbers of switched memory B cells and plasmablasts, implying a possible skewness toward isotype-switched phenotypes, at least in the two patients analyzed. This finding highlights the importance of B cell subsets analysis in PAD patients, as they are a heterogeneous population. In this study, patients did not show major complications usually associated with PAD, such as autoimmunity of malignancies, and accordingly, B cell subsets associated with B cell activation and anergic such as “IgMonly” memory B cells or CD21low B cells were not found alternated in the majority of the patients. Moreover, contrary to the reduction in absolute counts in all B cell subsets in CVID patients found in other studies, CVID patients in our study only showed an absolute reduction in some subsets (aside from the four subjects with less than 1% B cells) [9].

Euroclass and Driessen et al. classifications are important because they can potentially modify how the patients are monitored longitudinally over time, with smB-Trnorm and smB-21norm being the patients with the most probability to develop any non-infectious complication such as granulomas, autoimmunity and lymphoproliferation [12], but are also important as a starting point to further study the precise determination of the molecular mechanism(s) driving the disease in these patients, which would provide information for the understanding of the disease and the development of better treatments [25]. Finally, the purpose of including the pulmonary complications (PC) group was to control B cell subsets’ alterations due to bronchiectasis or recurrent pneumonia, as these pathologies could cause alterations within the immune system [26].

In conclusion, PAD participants showed alterations in lymphocytes’ populations and B cell subsets, especially in switched memory B cells, as reported commonly for CVID patients. Furthermore, the classification of these patients revealed different probable pathophysiological mechanisms of their disease, suggesting a heterogeneous population of PAD patients treated in Southwest Colombia. However, the number of PAD participants enrolled in this study is not optimal for statistical analysis due to the high complexity and heterogeneity of PAD disorders and no possible associations between the different classification systems and disease complications can be made. Therefore, studies with more patients are needed in Colombia, as PAD patients are currently understudied in the country.

### Clinical Implications/Future Directions

Results from this study are a basis for future studies about the classification and characterization of patients with predominantly antibody deficiencies in Colombia. A detailed immune response evaluation and large sample numbers are required for further determination of the population parameters for an improvement in the clinical management of these diseases.

## Figures and Tables

**Table 1:** Sociodemographic data of the participants. PC: Pulmonary complications group; HD: healthy donor group. Other PAD: patients with selective IgM or IgA deficiency or unclassified antibody deficiency.

Parameter	CVID	Other PAD	PC	HD
*n*	9	7	20	20
Age, *n* (%)				
15–29	4 (44)	1 (14)	6 (30)	7 (35)
30–39	4 (44)	1 (14)	5 (25)	6 (30)
40–50	0	2 (29)	2 (10)	5 (25)
>50	1 (11)	3 (43)	7 (35)	2 (10)
Gender, *n* (%)				
Male	7 (78)	1 (14)	9 (45)	8 (40)
Female	2 (22)	6 (86)	11 (55)	12 (60)
Ethnicity, *n* (%)				
Mestizo	7 (78)	5 (71)	15 (75)	17 (85)
Black	2 (22)	2 (29)	4 (20)	1 (5)
Indigenous	0	0	1(5)	0
White	0	0	0	2 (10)
Bronchiectasis, *n* (%)	0	1 (14)	6 (30)	-
Recurrent pneumonia, *n* (%)	3 (33)	3 (43)	7 (35)	-
Bronchiectasis and Recurrent pneumonia, *n* (%)	6 (67)	3 (43)	7 (35)	-
Exposure to biomass, *n* (%)				
Yes	1 (11)	2 (29)	4 (20)	-
No	8 (89)	5 (71)	16 (80)	-
Exposure to wood smoke, *n* (%)				
Yes	0	1 (14)	7 (35)	-
No	9 (100)	6 (86)	13 (65)	-
Exposure to tobacco smoke, *n* (%)				
Yes	3 (33)	3 (43)	6 (30)	-
No	6 (67)	4 (57)	14 (70)	-

**Table 2:** B cell subsets frequency and classification of PAD participants. Absolute frequency (cells/µL) is shown between parentheses.

Group	Age (Years)	Dx Delay (Years)	Pneumonia Episodes	Ig Treatment	Other Manifestations Diagnosed	CD4:CD8	B Cells	Total Memory B Cells	SM B Cells	UsM B Cells	Naive CD5-B Cells	Transitional B Cells	EUROclass	Driessen et al.
PAD														
CVID-1	56	11	3	Yes	Skin abscesses	0.4	<1 *	0.5 *	0.1 *	0.21 *	15 *	42 ~	B-	-
CVID-2	30	2	>5	Yes	Viral Hepatitis	0.6	12 (430.8)	8 (34.4)	1.3 (5.7) *	9.5 (40.8) *†	58.9 (253.7)	3.1 (13.1)	smB+21norm	NA
CVID-3	31	14	>10	No	sinusitis	8	<1 (2.9) *†	38 (1.1) †	23.5 (0.7) †	1.1 (0) *†	29.7 (0.9) †	2.2 (0.1) †	B-	Pattern 1
CVID-4	37	6	3	Yes	-	0.5	3.5 (90.2) *	15.7 (14.2) †	0.11 (0.1) *†	15.7 (14.2)	49.7 (44.8)	6.8 (6.1)	smB-Trnorm smB-21lo	Pattern 4
CVID-5	23	6	>3	Yes	Otitis, sinusitis	0.3	2.7 (77.5) *†	19.5 (15.1) †	3.4 (2.7) *†	12.1 (9.4) †	49.5 (38.4)	2.1 (1.6)	smB+21norm	Pattern 3
CVID-6	24	11	2	Yes	Diarrhea, ITP, AD, Sjögren syndrome	0.8	17.4 (819.5)	5.09 (41.7) *	0.5 (4.2) *†	6.7 (54.7)	42.4 (347.5)	7.9 (64.3) ~	smB-Trnorm smB-21lo	NA
CVID-7	16	6	6	Yes	-	1.4	<1 (0.2) *†	35.8 (0.1) †	0 (0) *†	0 (0) *†	2.6 (0) *†	0 (0) *†	B-	Pattern 1
CVID-8	36	9	>10	Yes	Kernicterus, Hypoacousis, otitis, asthma, gastritis, AD	1.9	14 (380.8)	14.9 (56.7)	7.6 (28.8)	5.1 (19.5)	60.1 (228.9)	2.4 (9.1)	smB+21norm	Pattern 5
CVID-9	21	2	>5	Yes	Sinusitis, nephrotic syndrome, gastroesophageal reflux	0.8	<1 (4.3) *†	0.1 (0) *†	0.05 (0) *†	0 (0) *†	0 (0) *†	0.2 (0) *†	B-	Pattern 1
SIgAD-1	34	17	>10	Yes	Esophageal candidiasis	1.1	9.3 (189.8)	1.18 (2.2) *†	0.09 (0.2) *†	2.8 (5.2) *†	59.6 (59.6)	6 (11.4)	smB-Trnorm smB-21norm	Pattern 3
SIgAD-2	63	28	>10	No	Gastritis, asthma	2.1	13.1 (305.2)	19.6 (59.8)	14.2 (43.3)	4.1 (12.4) ~	46.8 (46.8)	4 (12.2)	smB+21norm	Pattern 5
SIgAD-3	50	5	0	No	Gastritis, asthma	0.8	11.3 (340.1)	8.6 (29.4) *†	3.8 (13) *†	2 (6.8) *†	70.6 (70.6) ~	1.4 (4.7)	smB+21norm	Pattern 3
SIgAD-4	48	15	3	No	Gastritis, asthma, sinusitis	1.9	10.9 (170)	19 (32.3)†	3.2 (5.4) *†	15.7 (26.7)	59.1 (59.1)	1.2 (2) †	smB+21norm	Pattern 1
SIgMD-1	64	28	4	No	Asthma, hypothyroidism	4.7	10 (214)	27.9 (59.7)	24.5 (52.4)	5.3 (11.3) ~~	40.3 (86.2)	2.3 (5) *†	smB+21norm	NA
SIgMD-2	64	57	4	No	Asthma	1.9	4.6 (176.3) *	44.5 (78.5) ~	36.2 (63.8) ~	3.8 (6.6) ~	30.2 (53.2) †	0.3 (0.6) *†	smB+21norm	NA
UAD	15	13	>15	No	Asthma, sinusitis, AD, absent corpus callosum	2.3	7.6 (175.7)	16.4 (28.8) †	4.6 (8) *†	6.9 (12.1) †	43.1 (75.7)	6.4 (11.3)	smB+21norm	Pattern 3
HD	36 (28–42)	-	-	-	-	2.1	14 (290)	27.7 (72.2)	15.5 (43)	9.9 (21.7)	35.9 (102.2)	3 (7.2)	-	-
PC	39 (29–54)	-	4 (3–10)	No	-	1.8	9.2 (242)	20.5 (24.9)	11.3 (16.7)	6.7 (13.7)	50 (109)	3 (9)	-	-

AD: Atopic dermatitis; ITP: immune thrombocytopenic purpura; SM: switched memory; UAD: unclassified antibody deficiency; UsM: unswitched memory. * Reduction in relative frequency; † reduction in absolute frequency; ~ increase in relative frequency; ~~ increased relative and absolute frequency.

## Data Availability

Flow cytometry raw data or other encrypted relevant data could be available upon request to corresponding author.

## References

[1] Modell V., Orange J.S., Quinn J., Modell F. (2018). Global report on primary immunodeficiencies: 2018 update from the Jeffrey Modell Centers Network on disease classification, regional trends, treatment modalities, and physician reported outcomes. Immunol. Res..

[2] Bousfiha A., Jeddane L., Picard C., Al-Herz W., Ailal F., Chatila T., Cunningham-Rundles C., Etzioni A., Franco J.L., Holland S.M. (2020). Human Inborn Errors of Immunity: 2019 Update of the IUIS Phenotypical Classification. J. Clin. Immunol..

[3] Tangye S.G., Al-Herz W., Bousfiha A., Chatila T., Cunningham-rundles C. (2020). Human Inborn Errors of Immunity: 2019 Update on the Classification from the International Union of Immunological Societies Expert Committee. J. Clin. Immunol..

[4] Durandy A., Kracker S., Fischer A. (2013). Primary antibody deficiencies. Nat. Rev. Immunol..

[5] Baumann U., Routes J.M., Soler-Palacín P., Jolles S. (2018). The lung in primary immunodeficiencies: New concepts in infection and inflammation. Front. Immunol..

[6] Ramzi N., Jamee M., Bakhtiyari M., Rafiemanesh H., Zainaldain H., Tavakol M., Rezaei A., Kalvandi M., Zian Z., Mohammadi H. (2020). Bronchiectasis in common variable immunodeficiency: A systematic review and meta-analysis. Pediatr. Pulmonol..

[7] Chalmers J.D., Chang A.B., Chotirmall S.H., Dhar R., McShane P.J. (2018). Bronchiectasis. Nat. Rev. Dis. Prim..

[8] ESID Registry Working Party (2019). ESID Registry—Working Definitions for Clinical Diagnosis of IEI. https://esid.org/Working-Parties/Registry-Working-Party/Diagnosis-criteria.

[9] Al Kindi M., Mundy J., Sullivan T., Smith W., Kette F., Smith A., Heddle R., Hissaria P. (2012). Utility of peripheral blood B cell subsets analysis in common variable immunodeficiency. Clin. Exp. Immunol..

[10] Warnatz K., Denz A., Dräger R., Braun M., Groth C., Wolff-Vorbeck G., Eibel H., Schlesier M., Peter H.H. (2002). Severe deficiency of switched memory B cells (CD27+IgM-IgD-) in subgroups of patients with common variable immunodeficiency: A new approach to classify a heterogeneous disease. Blood.

[11] Piqueras B., Lavenu-Bombled C., Galicier L., Bergeron-Van Der Cruyssen F., Mouthon L., Chevret S., Debré P., Schmitt C., Oksenhendler E. (2003). Common variable immunodeficiency patient classification based on impaired B cell memory differentiation correlates with clinical aspects. J. Clin. Immunol..

[12] Wehr C., Kivioja T., Schmitt C., Ferry B., Witte T., Eren E., Vlkova M., Hernandez-Gonzalez M., Detkova D., Bos P.R. (2008). The EUROclass trial: Defining subgroups in common variable immunodeficiency. Blood.

[13] Driessen G.J., Van Zelm M.C., Van Hagen P.M., Hartwig N.G., Trip M., Warris A., De Vries E., Barendregt B.H., Pico I., Hop W. (2011). B-cell replication history and somatic hypermutation status identify distinct pathophysiologic backgrounds in common variable immunodeficiency. Blood.

[14] Vélez A.C., Castaño D.M., Gómez R.D., Orrego J.C., Moncada M., Franco J.L. (2015). Inmunodeficiencia común variable: Caracterización clínica e inmunológica de pacientese identificación de subgrupos homogéneoscon base en la tipificación de subpoblaciones de linfocitos B. Biomédica.

[15] Pasteur M.C., Bilton D., Hill A.T. (2010). British thoracic society guideline for non-CF bronchiectasis. Thorax.

[16] Kaplan K.A., Beierle E.A., Faro A., Eskin T.A., Flotte T.R. (2006). Recurrent pneumonia in children: A case report and approach to diagnosis. Clin. Pediatr..

[17] Van Dongen J.J.M., Van Der Burg M., Kalina T., Perez-Andres M., Mejstrikova E., Vlkova M., Lopez-Granados E., Wentink M., Kienzler A.-K., Philippé J. (2019). EuroFlow-Based Flowcytometric Diagnostic Screening and Classification of Primary Immunodeficiencies of the Lymphoid System. Front. Immunol..

[18] Bateman E.A.L., Ayers L., Sadler R., Lucas M., Roberts C., Woods A., Packwood K., Burden J., Harrison D., Kaenzig N. (2012). T cell phenotypes in patients with common variable immunodeficiency disorders: Associations with clinical phenotypes in comparison with other groups with recurrent infections. Clin. Exp. Immunol..

[19] Wong G.K., Huissoon A.P. (2016). T-cell abnormalities in common variable immunodeficiency: The hidden defect. J. Clin. Pathol..

[20] Lee J., Kuchen S., Fischer R., Chang S., Lipsky P.E. (2009). Identification and Characterization of a Human CD5 + Pre-Naive B Cell Population. J. Immunol..

[21] Sanz I., Wei C., Jenks S.A., Cashman K.S., Tipton C., Woodruff M.C., Hom J., Lee F.E.H. (2019). Challenges and opportunities for consistent classification of human b cell and plasma cell populations. Front. Immunol..

[22] Isnardi I., Ng Y.S., Menard L., Meyers G., Saadoun D., Srdanovic I., Samuels J., Berman J., Buckner J.H., Cunningham-Rundles C. (2010). Complement receptor 2/CD21- human naive B cells contain mostly autoreactive unresponsive clones. Blood.

[23] Celiksoy M.H., Yildiran A. (2016). A comparison of B cell subsets in primary immune deficiencies that progress with antibody deficiency and age-matched healthy children. Allergol. Immunopathol..

[24] Carsetti R., Rosado M.M., Donnanno S., Guazzi V., Soresina A., Meini A., Plebani A., Aiuti F., Quinti I. (2005). The loss of IgM memory B cells correlates with clinical disease in common variable immunodeficiency. J. Allergy Clin. Immunol..

[25] Stuchlý J., Kanderová V., Vlková M., Heřmanová I., Slámová L., Pelák O., Taraldsrud E., Jílek D., Králíčková P., Fevang B. (2017). Common Variable Immunodeficiency patients with a phenotypic profile of immunosenescence present with thrombocytopenia. Sci. Rep..

[26] King P.T., Hutchinson P., Holmes P.W., Freezer N.J., Bennett-Wood V., Robins-Browne R., Holdsworth S.R. (2006). Assessing immune function in adult bronchiectasis. Clin. Exp. Immunol..

